# Mechanical Hyperalgesia but Not Forward Shoulder Posture Is Associated with Shoulder Pain in Volleyball Players: A Cross-Sectional Study

**DOI:** 10.3390/jcm11061472

**Published:** 2022-03-08

**Authors:** Daniel Pecos-Martín, Sergio Patiño-Núñez, Jessica Quintero-Pérez, Gema Cruz-Riesco, Cintia Quevedo-Socas, Tomás Gallego-Izquierdo, Hector Beltran-Alacreu, Josué Fernández-Carnero

**Affiliations:** 1 Physiotherapy and Pain Group, Department of Physical Therapy, University of Alcalá, 28871 Madrid, Spain; daniel.pecos@uah.es (D.P.-M.); tomas.gallego@uah.es (T.G.-I.); 2 Departamento de Fisioterapia, Medicina y Ciencias Biomédicas, Universidad de A Coruña, Campus de Oza s/n, 15006 A Coruña, Spain; sergio.patino@udc.es; 3 Licenciatura de Fisioterapia, Facultad de Medicina, Benemérita Universidad Autónoma de Puebla, Puebla 72410, Mexico; jessquin09@hotmail.com; 4 Research Institute of Physiotherapy and Pain, University of Alcalá, 28805 Madrid, Spain; gemcruz44@gmail.es (G.C.-R.); cintiaquevedo1999@gmail.es (C.Q.-S.); 5 Toledo Physiotherapy Research Group (GIFTO), Faculty of Physical Therapy and Nursing, Universidad de Castilla-La Mancha, 45071 Toledo, Spain; 6 Department of Physical Therapy, Occupational Therapy, Rehabilitation and Physical Medicine, Rey Juan Carlos University, 28922 Alcorcón, Spain; josue.fernandez@urjc.es; 7 La Paz Hospital Institute for Health Research (IdiPAZ), 28029 Madrid, Spain; 8 Grupo Multidisciplinar de Investigación y Tratamiento del Dolor, Grupo de Excelencia Investigadora, URJC-Banco de Santander, 28922 Madrid, Spain; 9 Motion in Brains Research Group, Institute of Neuroscience and Movement Sciences (INCIMOV), Centro Superior de Estudios Universitarios La Salle, Universidad Autonóma de Madrid, 28023 Madrid, Spain; 10 Grupo de Investigación de Dolor Musculoesqueletico y Control Motor, Universidad Europea de Madrid, 28670 Villaviciosa de Odón, Spain

**Keywords:** forward shoulder angle, pectoralis minor length, mechanical hyperalgesia, volleyball

## Abstract

Shoulder antepulsion, altered scapular kinematics and imbalance of muscle activity are commonly associated with shoulder pain. This study aimed to observe if there is an association between the forward shoulder angle (FSA) and the pectoralis minor length index (PMI) in volleyball players with and without shoulder pain. Furthermore, this study observed if there is an association between shoulder posture and upper limb mechanical hyperalgesia in volleyball players with and without shoulder pain. Methods: a cross-sectional study was conducted in the Physiotherapy and Pain Research Center in Alcalá de Henares (Spain). A total of 56 volleyball players met the inclusion criteria and agreed to enter the study. Subjects were divided into two groups: shoulder pain group (SPG) and control group (without pain). The following measurements of the dominant sides of the players were collected: FSA, PMI, and pressure pain threshold (PPT) in serratus anterior, lower trapezius, infraspinatus, teres minor, upper trapezius, levator scapulae, pectoralis major, radial nerve, cubital nerve, and median nerve. Results: The Spearman’s Rho revealed no significant correlations were found between FSA and PMI. Moreover, Spearman’s Rho test revealed in the SPG a negative moderate correlation between FSA and Infraspinatus-PPT (Rho = −0.43; *p* = 0.02); FSA and levator scapulae-PPT (Rho = −0.55; *p* < 0.01); FSA and pectoralis major-PPT (Rho = −0.41; *p* = 0.02); PMI and cubital nerve-PPT (Rho = −0.44; *p* = 0.01). Conclusions: No association was found between the forward shoulder angle and the pectoralis minor index in volleyball players with and without shoulder pain. There is a moderate negative association between shoulder forward angle and muscle mechanical hyperalgesia in volleyball players with shoulder pain, but no such associations were found in volleyball players without shoulder pain. Treatment of the infraspinatus, levator scapulae, pectoralis major, and pectoralis minor muscles could improve shoulder pain and ulnar nerve mechanosensitivity.

## 1. Introduction

Overuse or repetitive motion injuries are very common in sports [1]. This type of injury can be a major source of disability and pain and can leave players unable to engage in physical activity or competition. There is little evidence on the real impact and severity in sport, due to the methodological difficulties involved in recording them [1]. Volleyball is one of the sports affected by overuse injuries [2,3,4]. The biomechanics of the different movements involved in volleyball, in particular the spike and serve, together with the anatomy of volleyball players coincide with the most prevalent overuse injuries, the shoulder region (19.0 ± 11.2%) and the spine (16.8 ± 9.7%) [3,4]. These structures are subjected not only to repetition of movement but also to high torsional values and amplitude of movement in short periods of time [3].

The assessment of posture and biomechanics is a useful clinical tool in shoulder pain. Shoulder antepulsion, also known as “rounded shoulder”, is characterized by a position in which the scapula rotates downwards and remains tilted forward, increasing cervical lordosis and upper thoracic kyphosis [5]. It has been previously published that postural or biomechanical alterations such as forward head position, shoulder antepulsion, altered scapular kinematics, and imbalance of muscle activity are associated with shoulder pain [6]. This could lead to the development of pain depending on the tolerance and adaptive capacity of the central nervous system [7]. In addition, there is evidence that a high percentage of patients with non-specific arm pain have their shoulders in antepulsion and head forward (78% and 71%, respectively) [6,8].

Loss of activity of the lower trapezius and serratus anterior, a marked thoracic kyphosis and the anatomy of the scapula itself can cause the shoulder antepulsion. In addition, tension of the pectoralis minor [5], which, together with the downward displacement of the coracoid process, may affect the gliding of the brachial plexus cords. Complete scapular protraction (due to its junctions with soft tissues and surrounding structures) may reduce the space between the clavicle and the first rib, restricting nerve gliding, and as a result of a combination of movements of structures of the shoulder girdle itself, anterior displacement of the humeral head may occur [9]. This can lead to some postural alterations such as an antepulsion of the shoulder can alter the mechanosensitivity of different tissues, thus decreasing their tolerance to mechanical stress even if it does not provoke a nociceptive response [10]. A recent study adds data on this association by concluding that individuals with shoulder impingement syndrome had a greater thoracic kyphosis and less extension movement than age- and gender-matched healthy controls [11]. For all these reasons, more studies are needed in the sports population to observe whether these types of associations or relationships exist and what clinical implications they may have.

Currently there are no studies that correlate the forward shoulder position with photometry with the shortening of the pectoralis minor in any type of population. Nor has it been studied whether these postural alterations are related to pain and mechanical hyperalgesia in volleyball players.

For the above reasons, the objectives of this study are as follows:

1. To observe the differences in upper limb posture and mechanical hyperalgesia between volleyball players with and without shoulder pain;
2. To observe if there is an association between the forward shoulder angle and the pectoralis minor index in volleyball players with and without shoulder pain;
3. To observe if there is an association between shoulder posture and upper limb mechanical hyperalgesia in volleyball players with and without shoulder pain.

## 2. Materials and Methods

This research was a cross-sectional study conducted according to the Strengthening the Reporting of Observational studies in Epidemiology (STROBE) statement [12], and following the declaration of Helsinki. The study protocol received approval from the Research Ethics Committee of University CEU Cardenal Herrera from Valencia (CEI16/0112). All the participants agreed to participate and signed an informed consent form. The study was conducted at the Physiotherapy and Pain Research Center in Alcalá de Henares (Spain).

### 2.1. Participants

Recruitment was carried out through an invitation to participate in the study was sent to the different volleyball sports clubs in the local area. Eligible participants were women and men from the age of 18 onwards and being a regular volleyball player (3 or more hours per week). Exclusion criteria included participants who has previous neck and shoulder injuries or surgery and were unable to read or speak in Spanish.

### 2.2. Procedure

Shoulder position was assessed in volleyball players with and without shoulder pain by determining the position of the humeral head and the length of the pectoralis minor muscle [6,13]. In addition, the mechanosensitivity of neuromuscular structures related to the upper quadrant of the evaluated subjects was assessed [14].

The assessments were performed by three physiotherapists independently and none of them knew the subject’s condition in relation to shoulder pain, this was done to ensure the simple blind. Study participants were randomly assigned to each assessment by choosing a ballot with a number on it. Subjects who selected number 1 were first assessed for shoulder position, subjects who selected number 2 were assessed for mechanosensitivity of the musculature, and subjects who selected number 3 were assessed for mechanosensitivity in the nerve trunks.

### 2.3. Outcome Measures

#### 2.3.1. Forward Shoulder Angle (Forward Shoulder Position)

Posture was assessed using a postural analysis software [14,15]. Markers were placed on the acromion and the spinous process of C7. Participants were placed in a standing position, sideways 40 cm from the wall, and instructed to maintain a natural resting position. A reflex camera (Nikon Model D5300 SLR, Tokyo, Japan) was placed on a tripod one meter high and three meters from the wall. One photograph was taken from the right side and one photograph from the left side.

The forward shoulder angle (FSA) was determined by calculating the angle formed by a vertical line passing posterior to the marker at C7 and a line connecting C7 and the acromion marker. Those participants who showed values equal to or greater than 52° were considered a forward shoulder position (FSP) [6]. This procedure has shown good reliability with an very high intraclass correlation coefficient (ICC) (0.89) [6]. The photographs were taken by a physiotherapist with more than 10 years of experience in the management of musculoskeletal pain and was blinded to the values obtained from the other assessments.

#### 2.3.2. Pectoralis Minor Length Measurements

Pectoralis minor (PM) length is expressed as the pectoralis minor index (PMI) which is calculated as PM length (cm)/subject height × 100. This normalization index is used to allow for the variety of soft tissue and body structure between subjects [16]. To measure the length of the PM muscle the reference points were the inferior medial angles of the coracoid process, and lateral to the sternocostal junction of the fourth rib on its underside. These landmarks have shown an ICC of 0.96 [16]. A digital caliper (Mitutoyo/200 mm, Kawasaki, Japan) was used to measure the distance between these two points. Measurement using a caliper has shown an ICC of 0.83 to 0.87 [17].

Participants were placed supine with both hands on the abdomen, with the shoulders slightly abducted, and in a relaxed elbow flexion position. The elbows were flexed to eliminate the passive influence of the biceps brachii muscle [18]. PMI assessment was performed by a second evaluator blinded to the other assessments values and with more than 10 years of experience in the management of musculoskeletal problems.

#### 2.3.3. Tissue Mechanosensitivity (Muscle and Nerve Trunks)

The degree of tissue mechanosensitivity was assessed by determining the pressure pain threshold (PPT) with algometry, i.e., quantitative assessment of the sensory perception of the mechanical stimulus [19]. The PPT was measured with a manual algometer (Wagner Force Dial, Model FDK20) which has a head of 1 cm^2^ and determines the pressure in kg/cm^2^ [19]. The pressure was increased by 1 kg per second until the subject indicated changes in pressure sensation. The assessor stopped applying pressure when the participant expressed pain. Three measurements were made at each location with a 30-s rest period in between. The mean value of the three measurements was used for statistical analysis. A third evaluator blinded to the other assessments measured the PPT of the following muscles: serratus anterior, lower trapezius, infraspinatus, teres minor, upper trapezius, levator scapulae, pectoralis major. The muscle was palpated to locate the most mechanosensitive point and perform the PPT measurement. Measurement of the PPT with an algometer has been shown to have good reliability with an ICC of 0.87 to 0.89 [19].

A fourth evaluator performed bilateral PPT measurements of the nerve trunks of the upper extremity: median nerve, radial nerve, and ulnar nerve. The nerves were evaluated at the locations described by Sterling et al. [19], which have shown good reliability with an ICC of 0.92 to 0.97. The median nerve identified in the ulnar fossa inside the tendon of the biceps muscle. The radial nerve was located in the lateral intermuscular septum between the medial and lateral head of the triceps brachii muscle, and the ulnar nerve was located in the ulnar canal of the elbow. Both evaluators had more than 10 years of experience in the management of musculoskeletal system alterations.

#### 2.3.4. Pain Intensity

Shoulder pain was assessed using a visual analog scale (VAS). The subject with pain indicated what their pain was on a 10-cm line where 0 represented no pain and 10 the worst pain imaginable. This tool has been shown to be reliable with an ICC of 0.94 [20]. The VAS measure was expressed in cm. Participants with more than 0 cm in the VAS were placed in the shoulder pain group (SPG), the rest of participants were placed in the control group (CG).

### 2.4. Sample Size

Sample size and power calculations were performed with an appropriate software (G*Power 3.1) [21]. This study was based on a model of correlations, and the FSA was the primary outcome, with an effect size of 0.75. Given an alpha level of 0.05 and a power of 0.80, two groups were generated with a total sample size of 50. The groups included shoulder pain and without pain (control) with a minimum of 25 participants per group.

### 2.5. Data Analysis

Statistical analysis was performed with the Statistical Package for the Social Sciences, version 28 (IBM Corporation, Armonk, NY, USA). The normality of the study variables was tested using the Shapiro–Wilk test. A normal distribution of the variables was not obtained in the Shapiro–Wilk test (*p* < 0.05). Qualitative variables are presented as an absolute value and the percentage of the relative frequency [*n* (%)]. Continuous variables are represented as median (1st and 3rd quartiles). All statistical tests were interpreted at a significance level of 5% (*p* < 0.05). To test the differences between groups for FSA, PMI, muscle PPT, and nerve PPT, the Mann–Whitney U test was performed to verify which ones entailed statistically significant differences. Finally, the correlations between the study variables were analyzed for each group with the Spearman’s Rho test considering the results as 0.01 to 0.19 very low correlation, 0.2 to 0.39 low correlation, 0.4 to 0.69 moderate correlation, 0.7 to 0.89 high correlation, 0.9 to 0.99 very high correlation, and 1 large or perfect correlation.

## 3. Results

### 3.1. Participants and Descriptive Data

A total of 56 volleyball players met the inclusion criteria and agreed to enter the study, leaving a sample of 28 in the SPG group and 28 in the CG. The median age of the sample was 22.5 (19 and 24), with most of them being female *n* = 33 (58.9%). No statistically significant differences were found in the descriptive characteristics measured in both groups (*p* > 0.05). The descriptive data of the participants are shown in Table 1.

### 3.2. Comparison between Groups

The Mann–Whitney U test revealed no statistical differences between groups for FSA (*p* = 0.33) and for PMI (*p* = 0.29), see Table 2. On the other hand, significant statistical differences between groups for muscle PPT in Lower Trapezius (*p* = 0.019), Infraspinatus (*p* < 0.01), Teres Minor (*p* < 0.01), Upper Trapezius (*p* = 0.019), Pectoralis Major (*p* = 0.02), and for radial nerve PPT (*p* = 0.04), see Table 2.

### 3.3. Correlations

The Spearman’s Rho test revealed in the SPG a negative moderate correlations between FSA and Infraspinatus-PPT (Rho = −0.43; *p* = 0.02); FSA and Levator Scapulae-PPT (Rho = −0.55; *p* < 0.01); FSA and Pectoralis Major-PPT (Rho = −0.41; *p* = 0.02); PMI and Cubital Nerve-PPT (Rho = −0.44; *p* = 0.01); VAS and Upper Trapezius-PPT (Rho = −0.41; *p* = 0.02); VAS and Median Nerve-PPT (Rho = −0.51; *p* < 0.01). No significant correlations were found between posture measurements (FSA and PMI) and VAS, see Table 3.

No significant correlations were found according to the Spearman’s Rho test in the CG, see Table 4.

## 4. Discussion

This study is the first one that had explored the association of the forward shoulder angle and the pectoralis minor index in volleyball players. To summarize, the two main principal findings of this study are as follows:

1. No association was found between the forward shoulder angle and the pectoralis minor index in volleyball players with and without shoulder pain;
2. Results show that mechanical hyperalgesia is increased in players with shoulder pain versus those without, but there are no differences in forward shoulder posture or shortening of the pectoralis minor;
3. There is a moderate negative association between shoulder forward angle with muscle mechanical hyperalgesia (Infraspinatus, Levator Scapulae and Pectoralis Major); and the pectoralis minor index with the mechanical hyperalgesia of the cubital nerve in volleyball players with shoulder pain. No such associations were found in volleyball players without shoulder pain.

### 4.1. Posture

The results of this study appeals to the fact that shoulder position is not a key factor or a clear contributor to shoulder pain [11,22]. These results are in agreement with the findings of Ozunlu et al. [23] and Ribeiro et al. [23] Ozunlu et al. [23] showed that asymmetric scapular posture in volleyball players might be normal and not necessarily related to injury and Ribeiro A et al. [23] reported that scapular asymmetry may be normal and it should not be automatically considered as a pathological sign in throwing athletes. This situation has also been related to other areas of the body, such as the low back region, where there is no correlation between imaging tests and pain [24].

Precisely and related to PMI, several recently conducted studies concluded that there is no association between shoulder pain and function with the length of the pectoralis minor in patients with chronic shoulder pain [25,26]. This is consistent with our results although they are not performed in a sports population.

Although it has been found in another study that patients with forward shoulders have greater scapular internal rotation and less serratus anterior activity, these alterations are not associated with shoulder pain [6]. Therefore, although it has been proposed that the round shoulder posture may be related to shoulder pain and dysfunction because it alters the kinematics and muscle activity generating stressful situations in the shoulder, it seems that this relationship cannot always be established [27]. Perhaps clinical reasoning should be based more on dynamic observation of posture rather than static observation of posture [28].

It has been suggested that in the assessment of shoulder posture in patients with shoulder pain it could be interesting to observe how the change in shoulder posture affects the signs and symptoms of the patient with shoulder pain [29]. Lewis JS et al. showed that changing posture in patients with shoulder pain had positive effects on mobility and pain in these subjects [29]. A cohort study investigated people with shoulder pain and concluded that the best prognostic factor for these patients was the change of symptoms during the modification of scapula posture in the arm elevation movement [30]. Therefore, postural alteration would be clinically relevant when postural modification during the assessment of the patient with shoulder pain also modifies the patient’s signs and symptoms [28]. On the other hand, demonstrating to a patient that symptoms are modifiable could give the individual confidence to move and better adherence to treatment [31].

### 4.2. Mechanical Hyperalgesia

The relationship between posture and tissue mechanosensitivity has been studied previously. Although the mechanical stress suffered by the structures involved in a poor posture could produce an alteration of the sensitivity of these structures, it seems that the presence of pain plays an important role in the degree of mechanosensitization [7,32,33]. Martinez-Merinero P et al. [7], Rojas VEA et al. [32], and Pacheco J et al. [33], showed that mechanosensitivity of neck muscles and upper extremity nerve trunks is related to neck pain more than to forward head position. Similarly, a study conducted by Haik M et al. [34] in which they found that subjects with shoulder pain demonstrated mechanical hyperalgesia compared with healthy controls.

The results obtained in our study have shown that the mechanosensitivity of the assessed muscles is related to shoulder pain and not with rounded shoulder posture. The infraspinatus, levator scapulae, and pectoralis major muscles were shown to have greater mechanical hyperalgesia in volleyball players with shoulder pain. These data are consistent with the results of Hidalgo-Lozano et al. reporting that elite swimmers with shoulder pain showed significant lower PPT in levator scapulae, sternocleidomastoid, upper trapezius, and infraspinatus compared with healthy athletes [35]. Pain and altered mechanosensitivity could be a consequence of repetitive overhead movements. The infraspinatus, levator scapulae, and pectoralis major muscles can produce shoulder pain [36]. In particular, the infraspinatus muscle has been shown to be important in shoulder problems [37,38]. It is a structure involved in functional problems and shoulder pain [37,38]. During a sports competition, volleyball players with shoulder pain were treated for the infraspinatus muscle and the players were able to continue in the competition [37].

On the other hand, another interesting finding of this study was the relationship the pectoralis minor index with mechanical hyperalgesia of the ulnar nerve. The pectoralis minor index expresses the length of the pectoralis minor muscle [16]. The association between a decrease in pectoralis minor length and shoulder pain has been described in athletes [39]. And pectoral shortening as a factor associated with volleyball-related shoulder pain and dysfuntion [40]. During arm elevation, subjects with shortened pectoralis minor showed decreased external rotation/retraction and posterior tilting of the scapula [40]. Limitation of scapular movements could compromise the pectoralis minor space and increase mechanical stress on the ulnar nerve [40]. Increased mechanical stress on the ulnar nerve could lead to pain and other neurogenic alterations in the upper extremity [41].

Although the relationship between the pectoralis minor muscle and the ulnar nerve has not been studied to our knowledge, it could be a cause of shoulder pain in all overhead activities.

### 4.3. Clinical Implications

Establishing a definitive structural diagnosis for an athlete presenting with shoulder pain is a difficult process. There appears to be a poor correlation between orthopedic and imaging tests and symptoms related to shoulder pain. In this regard, postural deviations have been frequently cited as a cause of shoulder pain and disability. This study, like others, challenges this approach. Therefore, the importance of postural disturbances during the assessment of a subject with shoulder pain should be approached differently. The shoulder forward posture may not be related to shoulder pain.

On the other hand, it seems that the shoulder musculature could be one of the causes of pain in these patients. During spiking in volleyball there is a significant increase in activity and stress on the shoulder musculature [42]. Probably, repeated overhead movements may produce an increase in mechanical muscle hyperalgesia. The mechanical muscle hyperalgesia could translate into increased tension and onset of shoulder pain.

Treatment of the infraspinatus, levator scapulae, pectoralis major, and pectoralis minor muscles could improve shoulder pain and ulnar nerve mechanosensitivity.

The literature suggests that mechanical hyperalgesia is related to poorer muscle function, and this leads to poorer motor control recruitment [35,43,44,45]. In sport medicine, this can be key factor in both prevention and injury recovery. Therefore, the results of this study suggest that the shoulder forward posture or the pectoralis minor index does not appear to be associated with shoulder pain, but yes, the mechanical hyperalgesia of the shoulder complex musculature. Therefore, an assessment of mechanical hyperalgesia in athlete patients with shoulder pain is suggested before posture. We can therefore suggest that in the examination of these patients it would be advisable to pay attention to the signs and symptoms of pain processing rather than to assessing shoulder posture.

### 4.4. Limitations

One of the main limitations of this study is that it is a cross-sectional study can only demonstrate association and not causation. Longitudinal studies are now needed to determine the role of mechanical hyperalgesia and posture in the development of shoulder pain in volleyball players. In addition, one of the limitations due to the small sample size and high effect size established, is that smaller correlations may exist. The population size was small. Future studies with larger samples are needed to further confirm the current results.

## 5. Conclusions

No association was found between the forward shoulder angle and the pectoralis minor index in volleyball players with and without shoulder pain. There is a moderate negative association between shoulder forward angle and muscle mechanical hyperalgesia in volleyball players with shoulder pain, but no such associations were found in volleyball players without shoulder pain. Treatment of the infraspinatus, levator scapulae, pectoralis major, and pectoralis minor muscles could improve shoulder pain and ulnar nerve mechanosensitivity.

## Figures and Tables

**Table 1:** Characteristics of the groups. Values are median (first and third quartiles) and *n* (%).

	Shoulder Pain Group (*n* = 28)	Control Group (*n* = 28)	*p* Value
Age (years)	21.5 (20 and 23.75)	21.5 (18 and 26)	0.74
Sex (female *n*, [%])	19 [67.9%]	14 [50%]	-
Weight (kg)	63.5 (57 and 71.75)	67.5 (57.25 and 76.75)	0.66
Height (cm)	170 (166.25 and 183.25)	178 (167.75 and 180.75)	0.25
BMI	22.57 (20.44 and 23.71)	21.85 (20.83 and 23.32)	0.49
Dominant side (right *n*, [%])	26 [92.9%]	21 [75%]	-
VAS (0–10 cm)	6.25 (6 and 7)	-	-
Pain duration (months)	1 (0 and 6.75)	-	-
FSP (yes *n*, [%])	15 [53.6%]	15 [53.6%]	-

VAS, visual analogue scale; FSP, forward shoulder position.

**Table 2:** Measurements of the study. Values are median (first and third quartiles) and *n* (%).

		Shoulder Pain Group (*n* = 28)	Control Group (*n* = 28)	Mann–Whitney U Test (*p* Value)
FSA (degrees)		49 (42.25 and 56.5)	50.5 (48 and 57.5)	0.33
PMI (cm)		13 (11.88 and 13.98)	13.83 (12.25 and 14.51)	0.29
PPT (kg/cm^2^)				
	Serratus Anterior	2.35 (1.6 and 3.07)	2.72 (2.26 and 3.37)	0.059
	Lower Trapezious	2.52 (2.21 and 3.1)	3 (2.72 and 3.8)	0.019 *
	Infraspinatus	2.3 (2.02 and 2.85)	3 (2.6 and 4)	<0.01 **
	Teres Minor	2.25 (1.92 and 2.73)	2.7 (2.3 and 4)	<0.01 **
	Levator scapulae	2.2 (1.62 and 2.73)	2.75 (1.8 and 3.37)	0.06
	Upper Trapezius	2.12 (1.6 and 2.43)	2.42 (2.1 and 3.46)	0.019 *
	Pectoralis Major	2.15 (1.55 and 2.57)	2.7 (2 and 3.58)	0.02 *
	Median Nerve	2.4 (2.1 and 3.13)	2.77 (2.21 and 3.17)	0.4
	Radial Nerve	3 (2.18 and 3.67)	3.62 (2.61 and 4.03)	0.04 *
	Cubital Nerve	2.65 (2.46 and 3.47)	3.32 (2.52 and 4)	0.27

* *p* < 0.05, ** *p* < 0.01. FSA, forward shoulder angle; PMI, pectoralis minor index; PPT, pain pressure threshold.

**Table 3:** Spearman’s Rho correlations in Shoulder pain Group.

	PMI	VAS	Serratus Anterior	Lower Trapezious	Infraspinatus	Teres Minor	Levator Scapulae	Upper Trapezius	Pectoralis Major	Median Nerve	Radial Nerve	Cubital Nerve
FSA	0.12	−0.21	−0.36	−0.21	−0.43 *	−0.33	−0.55 *	−0.24	−0.41 *	−0.2	−0.14	−0.13
PMI	1	−0.002	−0.12	0.2	−0.02	−0.03	−0.11	0.14	−0.17	−0.09	−0.16	−0.44 *

* *p* < 0.05; FSA, forward shoulder angle; PMI, pectoralis minor index; VAS, visual analogue scale.

**Table 4:** Spearman’s Rho correlations in Control Group.

	PMI	VAS	Serratus Anterior	Lower Trapezious	Infraspinatus	Teres Minor	Levator Scapulae	Upper Trapezius	Pectoralis Major	Median Nerve	Radial Nerve	Cubital Nerve
FSA	−0.29	-	0.01	0.98	0.11	−0.08	−0.02	0.21	−0.2	0.06	0.05	0.02
PMI	1	-	0.11	−0.12	−0.03	−0.06	−0.27	−0.1	0.03	−0.11	0	−0.08

FSA, forward shoulder angle; PMI, pectoralis minor index; VAS, visual analogue scale.

## Data Availability

Data available on request due to privacy and ethical restrictions.
